# Robotic transperitoneal versus retroperitoneal approach for anterior renal mass nephron-sparing surgery

**DOI:** 10.1007/s11701-023-01798-2

**Published:** 2024-02-14

**Authors:** Nicholas J. Lanzotti, Michael Felice, Sarang Janakiraman, Owen Lewer, Christopher James, Jeffrey L. Ellis, Goran Rac, Hiten D. Patel, Gopal N. Gupta

**Affiliations:** Loyola University Medical Center; Loyola University Medical Center; Loyola University Medical Center; Loyola University Medical Center; Loyola University Medical Center; Loyola University Medical Center; Loyola University Medical Center; Loyola University Medical Center; Loyola University Medical Center

**Keywords:** Kidney cancer, robotic partial nephrectomy, retroperitoneal surgery

## Abstract

**Introduction:**

Robotic nephron-sparing surgery is traditionally performed via a transperitoneal (TP) approach. However, the retroperitoneal (RP) approach has gained popularity, particularly for posterolateral renal masses. The RP approach is associated with shorter operative time, less blood loss, and shorter length of stay, while preserving oncologic outcomes in selected masses. Here, we aim to assess the feasibility of the RP approach in excising anterior renal masses.

**Methods:**

Patients ≥18 years of age who underwent robotic nephron-sparing surgery for anterior renal masses were retrospectively identified (2008–2022). Baseline demographics, tumor characteristics, and peri-operative data were collected and characterized based on TP vs RP approaches. Wilcoxon rank sum test and Pearson’s chi-squared test were used to compare continuous and categorical variables, respectively.

**Results:**

216 patients were included – 178 (82.4%) underwent TP approach and 38 (17.6%) underwent RP approach. Baseline demographics, preoperative tumor size,_and renal nephrometry scores were similar. The RP approach was associated with shorter operative (150 vs 203 min, p<0.001) and warm ischemia time (12 vs 21 min, p<0.001), and less blood loss (20 vs 100 cc, p=0.002) (Table 1). The RP approach was associated with shorter length of stay (1 vs 2 days, p<0.001) and less total complications (5.3% vs 19.1%, p=0.038). Major complication (Clavien-Dindo Grade ≥3) rates were similar. There was no difference in positive surgical margin rates or pathologic characteristics.

**Conclusions:**

Robotic RP approach for nephron-sparing surgery is feasible for eligible anterior tumors and is associated with favorable perioperative outcomes with preserved negative surgical margin rates.

## Introduction

Renal surgery has undergone numerous iterative changes over the past three decades. The stepwise movement from an open approach to a laparoscopic or robotic one has allowed for shorter postoperative stays and lower estimated blood loss while preserving oncologic outcomes.^1^ Similarly, resections have, when appropriate, decreased in scope from radical nephrectomies to partial nephrectomies to tumor enucleations with resultant preservation of renal function as more renal parenchyma is left intact.^2, 3^ The approaches utilized to gain access to these lesions have also evolved over time, moving from a transperitoneal (TP) approach to a retroperitoneal (RP) one where appropriate. Benefits of the RP approach include ready access to the renal artery given its posterior position within the hilum, the ability to bypass entering the peritoneum and thus avoid potential adhesions or other surgical concerns therein, and the potential for the retroperitoneum to provide a degree of compression to any leakage of blood or urine.^4, 5^

Historically, the RP approach has been utilized principally to address posterolateral lesions as it has a fairly direct line of sight on these surfaces of the kidney.^6–9^ However, the scope of the RP approach has gradually expanded to include the extirpation of masses in other regions of the kidney. Takagi et al. compared TP and RP approaches when treating lateral tumors while Gu and colleagues investigated the same in the upper pole with findings in both studies showing lower estimated blood loss (EBL) and a shorter length of stay (LOS) in RP approach patients and similar complication rates between RP and TP approaches.^10, 11^

There remains, however, some resistance towards utilizing the RP approach in the treatment of anterior lesions. The TP approach has the benefit of having a more direct line of access to an anterior renal lesion, albeit with the aforementioned drawbacks of having to enter the peritoneum and not having ready access to the renal artery. Further investigation of the use of the RP approach in the treatment of anterior lesions is warranted to assess whether the benefits demonstrated in posterior and lateral lesions hold true for anterior lesions. The goal of this paper is, therefore, to characterize the usage of the RP approach versus the TP approach in the treatment of anterior renal lesions and compare oncologic and perioperative outcomes between the two approaches.

## Methods

**Figure 1:**
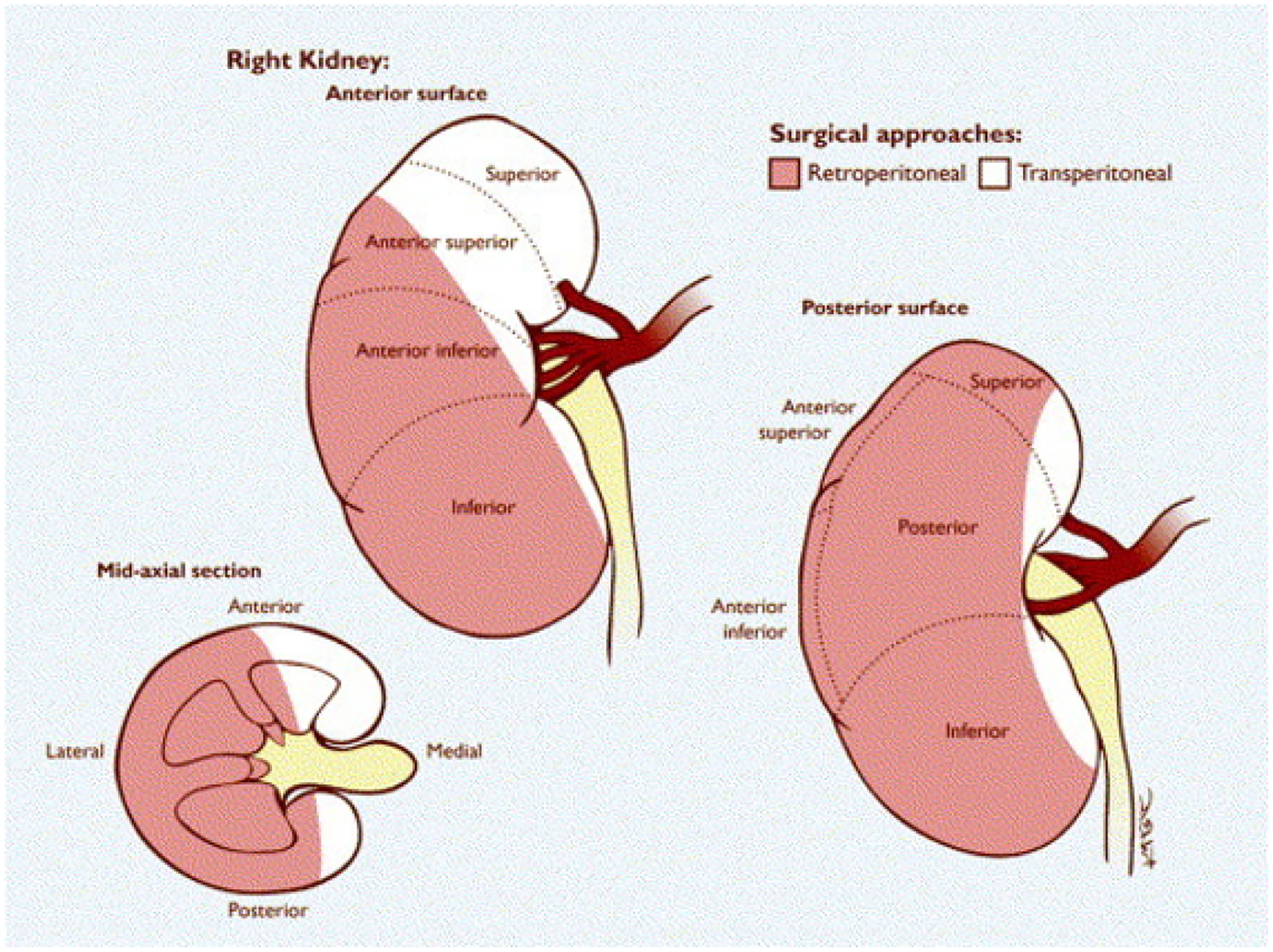
Diagram of eligible regions for RP approach. Reprinted with permission from Wright et al. JUrol 2005.

This study was deemed exempt from Institutional Review Board approval given its retrospective nature. We identified all patients ≥ 18 years of age with anterior renal masses who underwent robotic nephron-sparing surgery between 2008 and 2022 at Loyola University Medical Center with the goal of comparing outcomes for TP and RP. We captured baseline demographic characteristics, medical comorbidities (measured by Charlson Comorbidity Index), tumor information including RENAL nephrometry score, operative details, and postoperative course including renal function and imaging at time of outpatient follow-up. Preoperative imaging of each patient undergoing robotic nephron-sparing surgery was reviewed and assigned an anterior/posterior location. Additionally, all anterior tumors were then categorized as RP eligible or RP ineligible. Patients with anterior tumors undergoing RP were included in the RP cohort. Patients with anterior tumors included in the TP sample were limited to those with RP eligible tumors to ensure a similar anatomic distribution. Most tumor locations, except for predominantly upper pole anterior and centralized hilar tumors, were deemed eligible for the RP approach. Figure 1 depicts the anatomical regions of the kidney where the RP approach is deemed feasible at our institution. The RP approach utilized at our institution has been described previously.^5^

Surgical approach was selected in a non-randomized fashion at the discretion of the surgeon. Renal hilar clamping and renorrhaphy was performed at the discretion of the surgeon on a per case basis. Only malignant tumors were included for analysis of surgical margins. Complications were reported using the Clavien-Dindo classification. Major complications were considered Clavien-Dindo ≥ grade 3 complications. STATA version 15.0 (Stat Corp, College Station, TX) was used for statistical analysis. Continuous data was reported as median and interquartile range (IQR) and categorical data was reported as a number and percentage. Wilcoxon Rank Sum test was used to compare continuous data and the Pearson’s chi-squared test was used to compare categorical data.

## Results

216 patients qualified for inclusion – 178 (82.4%) underwent TP approach and 38 (17.6%) underwent RP approach. While the TP approach was utilized by four individual surgeons, all the patients that underwent the RP approach were treated by a single surgeon. Baseline patient demographics, comorbidities, and pre-operative evaluation are shown in Table 1. Baseline patient demographics and tumor characteristics were similar between the two groups. Patients who underwent the TP approach had significantly higher rates of prior abdominal surgery (p = 0.015). The median tumor size of the entire cohort was 2.7 cm (IQR 2–3.6 cm), and the majority of tumors were of low (RENAL 4–6) or intermediate (RENAL 7–9) complexity.

Table 2 depicts perioperative and short-term oncologic outcomes. There were significant differences in surgical technique between the two approaches. The RP approach utilized enucleation (86.8% vs 17.4%, p < 0.001) and off clamp resection (23.7% vs 8.4%, p < 0.001) more frequently. Operative time (150 vs 193 min, p < 0.001) and warm ischemia time (12 vs 21 min, p < 0.001) were shorter for the RP approach. Positive surgical margin rates (6.7% vs 4.1%, p = 0.68) were similar between approaches. There were fewer overall complications with the RP approach (5.3% vs 19.1%), but major complications were similar between approaches (0.0% vs 3.9%, p = 0.214). There were no significant differences in pathology or rates of chronic kidney disease stage 3 at 1 year of follow-up.

## DISCUSSION

Our study compares the perioperative and oncologic outcomes of treating anterior renal masses with either a TP or RP approach. These findings are notable in that the RP approach was found to have shorter operative time, warm ischemia time, LOS, and less EBL than TP. RP was also associated wither a low rate of overall complications although major complication rates were similar. This paper, therefore, adds to the growing body of evidence suggesting that the RP approach of treating renal lesions is both safe and efficacious. Furthermore, this paper is, to our knowledge, the first to explicitly focus on the feasibility of the RP approach in treating anterior lesions.

In that vein, there have been numerous studies in the last several years which have compared the RP approach favorably to the TP approach. Indeed, a meta-analysis limited to posterior lesions showed that RP partial nephrectomies were associated with shorter LOS while having similar operative time, EBL, positive surgical margin rates, morbidity, and warm ischemia time as compared to the TP approach.^9^ A second cumulative analysis which was not limited to posterior lesions showed that the RP approach is associated with shorter operative time, lower EBL, and shorter LOS than the TP approach.^12^

Our findings demonstrate a nearly one-hour shorter operative time when approaching the mass via an RP approach as compared to the TP approach. Shorter operative times for the RP approach are one of the more common benefits touted by other studies in the literature; however, there is heterogeneity in this trend.^7, 11 ,13–15^ Surprisingly, patients with a history of prior intra-abdominal surgery were significantly more likely to have surgery via the TP approach as opposed to the RP approach within our dataset (58.4% vs. 36.8%). While there was a statistically significant shorter warm ischemia time for the RP cases compared to the TP ones (12 vs. 21 min), that finding is less likely clinically significant.

Similar to others, our data shows that the RP approach is associated with less EBL than its TP counterpart^7, 10, 11, 14–17^ This was borne out in a recent meta-analysis, although other individual studies have found similar EBL between the two approaches.^9, 18, 19^

A 2020 paper investigating the use of the TP and RP approaches in the treatment of 566 patients from a single surgeon showed that the two groups of patients had similar Pentafecta (i.e., negative surgical margins, no postoperative complications, warm ischemia time ≤ 25 minutes, over 90% eGFR preservation, and no chronic kidney disease progression 1 year after surgery) achievement rates.^15^ In a sub-analysis assessing anterior lesions, they reported no difference in the T stage, RENAL score complexity, or histology for anterior lesions treated with TP or RP approach, which is similar to our experience. One factor that differentiates our experience from theirs, however, is the utilization of enucleation in the treatment of anterior masses. Indeed, in our cohort 86.8% of the lesions treated via the RP approach were enucleated, and 23.7% were performed off clamp. Both of these were statistically significant as compared to lesions that were addressed via the TP approach. Thus, our data suggests that the treatment of anterior lesions via the RP approach is not only safe but also provides the possibility for truly maximal nephron preservation via the reduction of devascularized parenchymal mass possible with these techniques.

There are limitations to our data that must be addressed. While the TP approach was utilized by several surgeons, only one utilized the RP approach which may lead to concern about the generalizability of these findings to general practice. The selective utilization of tumor enucleation and hilum clamping at surgeon discretion also introduces potential confounding into the data. The use of tumor enucleation was principally limited to patients with a definitive tumor pseudocapsule on pre-operative MRI and who have appropriately staged lesions without areas of subtle concern for invasion of the perinephric fat or evidence of local or vascular invasion.^20^ Nonetheless, the data presented here adds to the literature further evidence that the RP approach is another viable option in the armamentarium of urologic surgeons in the treatment of localized renal cancer even for anterior kidney lesions.

## Conclusions

Robotic RP approach for nephron sparing surgery is feasible for eligible anterior tumors. The RP approach for anterior tumors is associated with favorable perioperative outcomes with preserved negative surgical margin rates.

## Figures and Tables

**Table 1:** Patient baseline demographics

		Overall		Transperitoneal		Retroperitoneal		
		Median/N	IQR/%	Median/N	IQR/%	Median/N	IQR/%	p-value
**N**		216		178	82.4%	38	17.6%	
**Age (years)**		60.5	(52.1–67.7)	60.4	(52.8–67.7)	61.6	(49.1–69.2)	0.393
**Sex**	**Male**	126	58.3%	100	56.2%	26	68.4%	
	**Female**	90	41.7%	78	43.8%	12	31.6%	0.165
**Race**	**White**	162	75.0%	137	77.0%	25	65.8%	
	**Asian**	4	1.9%	2	1.1%	2	5.3%	
	**Black**	21	9.7%	18	10.1%	3	7.9%	
	**Hispanic**	26	12.0%	18	10.1%	8	21.1%	
	**Other**	2	0.9%	2	1.1%	0	0.0%	0.197
**Body Mass Index (kg/m^2^)**	**<25**	32	14.8%	25	14.0%	7	18.4%	
	**25–30**	68	31.5%	55	30.9%	13	34.2%	
	**30–35**	60	27.8%	50	28.1%	10	26.3%	
	**35+**	56	25.9%	48	27.0%	8	21.1%	0.808
**Prior Abdominal Surgery**	**Yes**	118	54.6%	104	58.4%	14	36.8%	
	**No**	98	45.4%	74	41.6%	24	63.2%	0.015
**Prior Kidney Surgery**	**Yes**	10	4.6%	9	5.1%	1	2.6%	
	**No**	206	95.4%	169	94.9%	37	97.4%	0.518
**Chronic Kidney Disease Stage ≥ 3**	**Yes**	45	20.8%	38	21.3%	7	18.4%	
	**No**	171	79.2%	140	78.7%	31	81.6%	0.687
**Charlson Comorbidity Index**	**0**	138	63.9%	116	65.2%	22	57.9%	
	**1**	46	21.3%	38	21.4%	8	21.1%	
	**2**	19	8.8%	13	7.3%	6	15.8%	
	**≥ 3**	13	6.0%	11	6.2%	2	5.3%	0.412
**Tumor Size (cm)**		2.7	(2–3.6)	2.8	(2–3.5)	2.55	(2–3.7)	0.796
**Tumor Laterality**	**Left**	100	46.3%	78	43.8%	22	57.9%	
	**Right**	116	53.7%	100	56.2%	16	42.1%	0.114
**Clinical T Stage**	**cT1a**	186	86.1%	152	85.4%	34	89.5%	
	**cT1b**	30	13.9%	26	14.6%	4	10.5%	0.509
**RENAL Nephrometry Score**	**Low (4 to 6)**	94	43.5%	76	42.7%	18	47.4%	
	**Intermediate (7 to 9)**	112	51.9%	94	52.8%	18	47.4%	
	**High (≥ 10)**	19	4.6%	8	4.5%	2	5.3%	0.829

TE = tumor enucleation; SPN = standard margin partial nephrectomy; IQR = interquartile range

**Table 2:** Peri-operative outcomes stratified by approach.

		Overall		Transperitoneal		Retroperitoneal		
		Median/N	IQR/%	Median/N	IQR/%	Median/N	IQR/%	p-value
**N**		216		178	82.4%	38	17.6%	
**Resection**	**Standard Margin**	152	70.4%	147	82.6%	5	13.2%	
	**Enucleation**	64	29.6%	31	17.4%	33	86.8%	<0.001
**Hilum Clamped**	**Yes**	192	88.9%	163	91.6%	29	76.3%	
	**No**	24	11.1%	15	8.4%	9	23.7%	0.007
**WIT (min)**		20	(12–27)	21	(14–28)	12	(8–20)	<0.001
**Surgical Margin**	**Negative**	166	95.4%	138	95.9%	28	93.3%	
	**Positive**	8	4.6%	6	4.1%	2	6.7%	0.680
**Pathologic Size (cm)**		2.7	(2.1–3.5)	2.7	(2.1–3.5)	2.6	(2–3.5)	0.316
**Pathologic T Stage**	**pT1a**	144	67.2%	119	66.9%	25	65.8%	
	**pT1b**	23	10.7%	20	11.2%	3	7.9%	
	**pT3a**	12	5.6%	10	5.6%	2	5.3%	
	**N/A**	35	16.4%	29	16.3%	8	21.1%	0.838
**Histology**	**Malignant**	176	81.9%	146	82.5%	30	79.0%	
	**Benign**	39	18.1%	31	17.5%	8	21.0%	0.787
**Histology**	**Clear Cell RCC**	120	55.6%	102	57.3%	18	47.4%	
	**Papillary Type 1**	26	12.0%	20	11.2%	6	15.8%	
	**Papillary Type 2**	2	0.9%	2	1.1%	0	0.0%	
	**Clear Cell & Papillary RCC**	11	5.1%	8	4.5%	3	7.9%	
	**Chromophobe RCC**	12	5.6%	10	5.6%	2	5.3%	
	**Cystic RCC**	1	0.5%	1	0.6%	0	0.0%	
	**Unclassified RCC/Other** ^**1**^	5	2.3%	4	2.2%	1	2.6%	
	**Angiomyolipoma**	13	6.0%	11	6.2%	2	5.3%	
	**Oncocytoma**	21	9.7%	16	9.0%	5	13.2%	
	**Other Benign**	5	2.3%	4	2.2%	1	2.5%	0.820
**ISUP Grade**	**1**	14	9.0%	9	7.0%	5	19.2%	
	**2**	105	67.7%	90	69.8%	15	57.7%	
	**3**	30	19.4%	24	18.6%	6	23.1%	
	**4**	6	3.9%	6	4.6%	0	0.0%	0.139
**Complication**	**Yes**	36	16.7%	34	19.1%	2	5.3%	
	**No**	180	83.3%	144	80.9%	36	94.7%	0.038
**Major Complication**	**Yes**	7	3.2%	7	3.9%	0	0.0%	
	**No**	209	96.8%	171	96.1%	38	100.0%	0.214
**Readmission**	**Yes**	14	6.5%	13	7.3%	1	2.6%	
	**No**	202	93.5%	165	92.7%	37	97.4%	0.288
**EBL (mL)**		100	(50–250)	100	(50–300)	20	(10–50)	0.002
**Operative Time**		193	(156–226)	203	(165–237)	150	(120–190)	<0.001
**Conversion**	**Yes**	3	1.4%	2	1.1%	1	2.6%	
	**No**	213	98.6%	176	98.9%	37	97.4%	0.471
**Length of Stay (days)**		2	(1–3)	2	(1–3)	1	(1–1)	<0.001
**CKD3 at 1 year**	**Yes**	54	29.0%	46	30.7%	8.0	22.2%	
	**No**	132	71.0%	104	69.3%	28.0	77.8%	0.316
